# Hypoxia inducible factors in cancer stem cells

**DOI:** 10.1038/sj.bjc.6605551

**Published:** 2010-01-26

**Authors:** J M Heddleston, Z Li, J D Lathia, S Bao, A B Hjelmeland, J N Rich

**Affiliations:** 1 Department of Stem Cell Biology and Regenerative Medicine, Lerner Research Institute, Cleveland Clinic Foundation, Cleveland 44195, OH, USA

**Keywords:** HIF, hypoxia, cancer stem cell

## Abstract

Oxygen is an essential regulator of cellular metabolism, survival, and proliferation. Cellular responses to oxygen levels are monitored, in part, by the transcriptional activity of the hypoxia inducible factors (HIFs). Under hypoxia, HIFs regulate a variety of pro-angiogenic and pro-glycolysis pathways. In solid cancers, regions of hypoxia are commonly present throughout the tissue because of the chaotic vascular architecture and regions of necrosis. In these regions, the hypoxic state fluctuates in a spatial and temporal manner. Transient hypoxic cycling causes an increase in the activity of the HIF proteins above what is typical for non-pathologic tissue. The extent of hypoxia strongly correlates to poor patient survival, therapeutic resistance and an aggressive tumour phenotype, but the full contribution of hypoxia and the HIFs to tumour biology is an area of active investigation. Recent reports link resistance to conventional therapies and the metastatic potential to a stem-like tumour population, termed cancer stem cells (CSCs). We and others have shown that within brain tumours CSCs reside in two niches, a perivascular location and the surrounding necrotic tissue. Restricted oxygen conditions increase the CSC fraction and promote acquisition of a stem-like state. Cancer stem cells are critically dependant on the HIFs for survival, self-renewal, and tumour growth. These observations and those from normal stem cell biology provide a new mechanistic explanation for the contribution of hypoxia to malignancy. Further, the presence of hypoxia in tumours may present challenges for therapy because of the promotion of CSC phenotypes even upon successful killing of CSCs. The current experimental evidence suggests that CSCs are plastic cell states governed by microenvironmental conditions, such as hypoxia, that may be critical for the development of new therapies targeted to disrupt the microenvironment.

Oxygen concentration is tightly regulated at the tissue and cellular levels because of its essential role in many biological processes. Hyperoxia can induce the formation of reactive oxygen species (ROS), which may cause genotoxic effects or cell death. In contrast, hypoxia can have extensive downstream transcriptional effects, such as activation of pro-apoptotic and pro-angiogenic pathways. Nonpathological cells have regulatory mechanisms to quickly respond to variations in oxygen tension by the hypoxia inducible factors (HIFs). Neoplastic cells employ oxygen-sensitive activity of the HIF proteins. The HIFs also have higher basal activity in the neoplastic compartment. However, this enhanced level of transcriptional activity is not fully understood. It can be partly attributed to the state of oxygenation within a tumour, which is not consistent and behaves in a cyclical nature. The flux in oxygen tension experienced by cancer cells is an effect of several tumour characteristics such as chaotic vasculature, poor oxygen diffusion across an ever-expanding tumour, and irregular blood flow. Several studies have shown that the constant acute cycling of hypoxic regions in a tumour leads to enhanced metastatic potential and HIF transcriptional activity beyond what is typical of healthy tissue (Cairns *et al*, 2001; Peng *et al*, 2006). Oxygenation status of tumour tissue cycles in both spatial and temporal manner. Studies in mice utilising window chamber imaging techniques demonstrated that hypoxic cycling occurs often in regions of microvasculature because of the instability of red blood cell flow through the vessels (Kimura *et al*, 1996). Consequently, this regional cycling results in fluctuating HIF activity, which has been shown to directly correlate to tumour radiation resistance (Moeller *et al*, 2004; Dewhirst *et al*, 2008). Recent experimental evidence has expanded on the role of hypoxia in cancer by demonstrating differential responses to hypoxia between heterogenic sub-populations within the tumour. One of these populations, termed cancer stem cells (CSCs), possess many phenotypic similarities to normal stem cells, such as having the ability to upregulate DNA repair kinases to evade radiation-induced genomic damage (Bao *et al*, 2006a). Although somewhat controversial, hypoxia has become increasingly related to the cancer stem cell phenotype. The role of HIFs in these cells has become an important focus in understanding tumour growth and survival (Hill *et al*, 2009).

## Stem-like cells in solid tumours

Virchow proposed the embryonic rest theory in cancer (Virchow, 1858) 150 years ago. In the early twentieth century several connections were made between cancer cells and less-differentiated normal cells. In a seminal work, the Dick laboratory isolated patient-derived stem-like leukaemia cells that were capable of initiating *de novo* leukaemia in SCID mice (Lapidot *et al*, 1994). They also demonstrated that myeloid leukaemia cells were organised as a hierarchy and originated from a primitive haematopoietic cell (Bonnet and Dick, 1997). Dick's studies in leukaemia were furthered by Clarke and colleagues (Al-Hajj *et al*, 2003) through application of the hierarchical principle to breast cancer. They discovered a subset of cells that could be prospectively enriched in their cultures by fluorescence-activated cell sorting (FACS) for surface markers typically associated with normal stem cells, CD44 and CD24 (Al-Hajj *et al*, 2003). The cells that expressed this marker pattern were shown to be more tumourigenic in SCID mice. In addition, tumours arising from this sub-population maintained the phenotypic heterogeneity present in the primary tumour, suggesting that the stem-like sub-population within breast cancer was primarily responsible for the formation of tumour and establishment of neoplastic heterogeneity. These cells, known as cancer stem cells, tumour initiating cells, or tumour propagating cells, utilise many unique survival techniques to propagate the tumour, some of which are similar to those found in normal somatic stem cells. Since this early work, several groups have demonstrated the presence of cancer stem cells in other solid tumours, including malignant glioma and colorectal cancer (Singh *et al*, 2003; Ricci-Vitiani *et al*, 2007).

**Figure 1:**
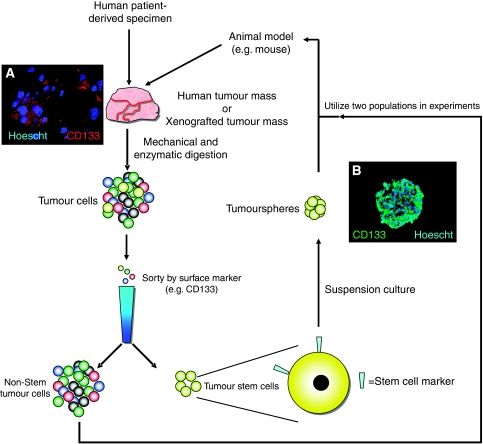
Enrichment of cultures for cancer stem cells allow for better study of their unique biology. In order to appropriately examine the biological significance of the cancer stem cell population, *in vitro* cultures must be enriched for this population before experimental investigation. Utilising animal models, such as immunocompromised mice, patient-derived cancer cells can be expanded for use in the laboratory. Following resection of the tumour from the patient, the mass is dissociated into single cells through a combination of mechanical and enzymatic digestion. Once the cells have recovered and are growing as single cells, they can be sorted based on surface marker expression. Experimental evidence has demonstrated that the cancer stem cell sub-population express a subset of genes that can act as markers for enriching cultures for the stem-like cancer cells (Singh *et al*, 2003). The present gold standard of these markers in glioma is the surface protein, CD133 (also known as Prominin-1). CD133 can be used in cell sorting to discern the cancer stem cell sub-population. Although little is known about its role in cancer biology, (**A**) CD133 expression has been observed on a sub-population of cells in the tumour tissue and (**B**) this expression is maintained in the cancer stem cell population *in vitro*. In (**A**) a subcutaneous tumour was resected out of a mouse, fixed in paraformaldehyde, and frozen. Sections of the tumour were cut and stained for CD133 (red) and nuclei (Hoescht 3342, blue). In (**B**) cancer stem cells enriched from glioblastoma multiforme were cultured in serum-free media until they formed spheroids (neurospheres). The cells were then fixed, frozen, and cut. The sections were stained for CD133 (green) and nuclei (Hoescht 3342, blue). This procedure allowed for direct staining of cells throughout the sphere. Once the tumour cells have been separated into cancer stem cell (CSC)-enriched and non-CSC populations, experiments can be carried out to elucidate the biological differences between the two populations. The most important way to test CSC biology is the ability of the cancer stem cells to form tumours that are phenotypic copies of the parental tumour. In the glioma system, one way to determine a cells tumourigenic capacity is direct intracranial implantation. In this way, the tumour formation capacity of cancer cells allows researchers to delineate the stem-like fraction of cells within a tumour.

Cancer stem cells contribute to tumour growth, maintainence, and recurrence after therapy through multiple mechanisms and networks. One important characteristic of these cells is their ability to restrict DNA damage sustained during radiation or chemotherapy by reduction of ROS and enhanced activity of DNA checkpoint kinases (Bao *et al*, 2006a; Diehn *et al*, 2009). By preventing DNA damage, the cancer stem cell population survives injury and can continue to propagate the tumour. CSC were also shown to be dependant on the Akt signalling pathways, as the cancer stem cell sub-population had increased sensitivity to chemical Akt inhibitors (Eyler *et al*, 2008). To enrich *in vitro* cultures for this population, sorting protocols were developed that took advantage of unique markers expressed by cancer stem cells when compared with the bulk of the tumour (Figure 1). Additional experimental evidence has demonstrated that one of the important roles the cancer stem cell population has in a tumour is in regulating tumour angiogenesis by vascular endothelial growth factor (VEGF) signalling.

Angiogenesis is essential for tumour survival and is the canonical downstream effect of HIF transcriptional activity. As cells grow and divide, the neoplastic compartment rapidly expands past the diffusion distance of oxygen in tissue (∼100 *μ*m). Large regions of the tumour become hypoxic because of this spatial limitation. The immediate cellular response (mediated by HIF proteins) is the influx of new vessels to provide appropriate oxygenation to the tumour tissue. However, vessel ingrowth cannot maintain proper tissue oxygenation when faced with the rapid cellular expansion of a tumour. This constant imbalance between cell and vessel growth causes a fluctuating oxygen state. These transient states of hypoxia are a common characteristic of solid tumours. Although normal oxygen tension in healthy tissue is approximately 7% oxygen (53 mmHg), this tension in a tumour can range from physiological (∼7%) to severe (<1%) oxygen. Severe hypoxia is less frequent but commonly found surrounding areas of necrosis, which is another common characteristic of solid tumours. The immediate molecular response to low oxygen is stabilisation of the HIF proteins. Hypoxia inducible factors stimulate the expansion and migration of endothelial cells into the tumour space, which allow new vessel growth from the existing vasculature structure surrounding the tumour. The formation of these vessels supplies the rapidly expanding tumour with nutrients and oxygen. However, the vasculature is very chaotic and does not transfer nutrients and oxygen efficiently (Baish and Jain, 1998). This is thought to be a consequence of the number of angiogenic signals coming from the neoplastic cell population, such as VEGF and the angiopoietins. However, until recently it was hypothesised that the majority of cells in the tumour were capable of regulating this angiogenic signal. It is now known that there is heterogenic expression of VEGF within a tumour. The cancer stem cell sub-population has been shown to be the primary regulator of angiogenesis in a tumour by the production of VEGF (Bao *et al*, 2006b). The chaotic vessel formation is another factor in the constant oxygen flux observed in solid tumours (Carmeliet and Jain, 2000). High frequency of cycling (acute) hypoxia correlates to lower patient survival. This sensitivity of tumour structure to oxygen status is driven by the HIF proteins in the cancer stem cell population.

## Oxygen sensitive HIF activity

The HIFs are transcription factors whose expression and stability is regulated by oxygen tension. They exist as a heterodimer, consisting of an alpha and beta subunit. There are three isoforms of the alpha subunit: HIF1*α*, HIF2*α* (also known as endothelial PAS-domain protein 1, EPAS1), and HIF3*α*. The HIF*β* isoforms, also known as aryl hydrocarbon receptor nuclear translocator (ARNT and ARNT2), are constitutively and ubiquitously expressed across many cell types (Maltepe *et al*, 1997; Keith *et al*, 2001). The present understanding of HIF regulation has described the majority of oxygen-controlled HIF regulation occurring on the alpha subunit, whereas the beta subunit is not sensitive to oxygen levels and is constitutively present in the nucleus. The HIF1*α* subunit is a basic helix-loop-helix protein whose structure and function is evolutionarily conserved between mice and humans (Iyer *et al*, 1998). Hypoxia inducible factor-1*α* has been well-studied and is ubiquitously expressed in normal tissue. Further studies characterized a second HIF*α* isoform as also being tightly regulated by oxygen tension. Since its initial discovery, HIF2*α* was demonstrated to have shared transcriptional targets with HIF1*α* such as VEGF, Tie-2, Ang2, and Flt1 (VEGF-R1). HIF1*α* and HIF2*α* also bind homologous target DNA-binding sequences (Lau *et al*, 2007). Despite their similarities, HIF2*α* expression was restricted to endothelial cells of vascular organs and had several unique transcriptional targets such as Oct4 and TGF*α*. These targets are outside the canonical pro-angiogenic hypoxic response, which suggests an important and specific role for HIF2*α* in regulating other cellular processes such as pluripotency. Little is known about the third HIF*α* isoform. Several splice variants of HIF3*α* have been shown to be a dominant-negative regulator of the other two alpha isoforms and has a limited expression pattern in the eye and the cerebellum. Some HIF3*α* isoforms are also thought to be direct transcriptional targets of HIF1*α* activity under hypoxia. Current studies are still unclear as to the primary function and regulatory mechanism through which HIF3*α* and its variants function (Makino *et al*, 2001; Maynard *et al*, 2003).

Certain disease states allow for the stabilisation of the HIF*α* subunit even in the presence of oxygen. One of the more well-known conditions is renal cell carcinoma (RCC). In RCC, there is a biallelic inactivation of the E3 ubiquitin ligase responsible for targeting the HIF*α* subunits for degradation. Renal cell carcinoma patient specimens have higher activity of HIF regulated pathways such as increased angiogenesis, altered glucose uptake and metabolism, and loss of growth control by mitogenic signals. HIF1*α* and HIF2*α* have unequal roles in RCC and HIF2*α* is more important for disease progression. Inhibition of HIF2*α* suppresses *in vivo* tumour growth (Kondo *et al*, 2003). This suggests that in a system where both HIF1*α* and HIF2*α* are stabilized and functional, HIF2*α* is critical to tumour growth and survival whereas HIF1*α* is not. HIF2*α* is stabilized at a wider range of oxygen tensions, ranging from severe hypoxia (<1% oxygen) to more physiologically relevant tension for tumours (2–5% oxygen) (Holmquist-Mengelbier *et al*, 2006). HIF2*α* can respond to a wider range of oxygen tensions to activate downstream transcriptional targets. The tumour would have the capability to adapt to an ever-changing microenvironment beyond the regions of most severe hypoxia. However, HIF1*α* also has a role in addition to HIF2*α* in promoting tumour growth. In solid tumours HIF1*α* has been linked to regulating oncogene activity through AKT and epidermal growth factor receptor (EGFR) pathways. The different roles and expression patterns of HIF1*α* and HIF2*α* demonstrate that their specificity is important for tumour propagation and survival. The HIFs have been demonstrated to be expressed in the cancer stem cell population, possibly promoting the stem-like phenotype and driving tumour growth.

### The role of HIFs in cancer stem cells

Although originally described in endothelial cells as regulators of migration and erythropoiesis, the HIF proteins have since been implicated in many cell processes. Specifically in cancer, they are critical for the ingrowth of vasculature to feed the rapidly expanding neoplastic compartment. As more is understood about the methods of tumour propagation, hypoxia has become a critical microenvironmental influence. Lending support to this theory is the recent evidence linking HIF expression in cancer stem cells to cell proliferation and tumour survival.

Early work on HIFs and cancer focused on HIF1*α* and demonstrated that the majority of tumour cells responded to hypoxia by stabilizing the HIF1*α* protein. In many cases HIF2*α* expression was not found or was no different between hypoxia and normoxia. The conclusion was drawn that HIF1*α*, not HIF2*α*, was responsible for the majority of the hypoxia response in cancer. However, recent experimental evidence from our laboratory has demonstrated differential protein and mRNA expression of HIF1*α* and HIF2*α* between the non-stem and cancer stem cell (Li *et al*, 2009). Further, this study elucidated that HIF2*α* was only significantly present in the cancer stem cell population. In comparison, HIF1*α* was present in both stem and non-stem tumour cell population and was only stabilized in more severe hypoxic conditions. This finding suggests differential roles of the two isoforms in cancer stem cells. Furthermore, several genes previously associated with the hypoxic response in normal cells were shown to have higher expression in the cancer stem cell sub-population, such as Glut1, Serpin B9, and VEGF (Bao *et al*, 2006b; Li *et al*, 2009). Following *in vivo* knockdown experiments, this study concluded that HIFs were required for cancer stem cell survival and tumour propagation. Cancer stem cells that had diminished HIF activity were unable to form tumours and had reduced survival *in vitro*. This suggests that HIFs has a much larger role in cancer stem cells than initially hypothesised. Current studies are demonstrating an increased importance of the HIF proteins in maintaining an undifferentiated phenotype (Jogi *et al*, 2002).

### HIFs promote a stem cell phenotype

Several reports have shown that hypoxia and HIFs are involved in maintaining a more stem-like state in normal tissue (Clarke and van der Kooy, 2009; Moreno-Manzano *et al*, 2009). Through signalling pathways like Notch and Oct4, the HIFs are being understood as crucial regulators of the stem cell phenotype (Gustafsson *et al*, 2005). Recent reports have demonstrated the ability of the hypoxic microenvironment to greatly potentiate the biological effect of Notch in adenocarcinoma of the lung (Chen *et al*, 2007). In tissues throughout the body, stem cells persist in specific anatomical locations, also referred to as niches. One example of these niches can be found in bone marrow where haematopoietic stem cell are known to reside in regions regulated by oxygen tension (Parmar *et al*, 2007). Growing evidence supports the hypothesis that stem cells residing in hypoxic niches rely on HIF activity to maintain their undifferentiated phenotype. It is interesting to note that the presence of hypoxic areas within a tumour also suggest possible niches for cancer stem cells (Gilbertson and Rich, 2007). The correlation of tumour hypoxia to poor patient outcome may be related to an increase in the presence of cancer stem cells. In the emerging field of induced pluripotency, a recent study by Yamanaka and colleagues (Yoshida *et al*, 2009) has shown that culture in hypoxia significantly improves the generation of iPS colonies following reprogramming. Current understanding of cancer stem cell biology and their similarities to somatic stem cells suggest that hypoxia and the HIFs may act to regulate the cancer stem cell phenotype.

**Figure 2:**
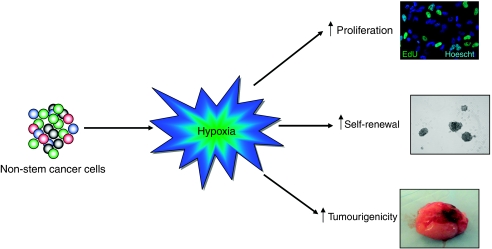
Hypoxia and the hypoxia inducible factors (HIFs) can promote the stem-like phenotype. Recent experimental evidence has demonstrated that HIFs have crucial roles in cancer. Following culture in low-oxygen conditions, the phenotype non-stem cancer cells were pushed to a more stem-like state. Several important characteristics of the cancer stem cell population, such as enhanced growth, self renewal (evidenced by spheroid formation), and tumourigenesis, have been shown to increase in the non-stem fraction of cancer cells following hypoxic culture (Heddleston *et al*, 2009). Non-stem cells increase their rate of proliferation above what is normally seen at 20% oxygen. This can be visualised by EdU retention (an analogue of BrDU). Hypoxia has also been shown to promote self-renewal, which can be measured by spheroid growth starting from a single cell. Non-stem cancer cells expressing constitutively active HIF2*α* protein have been demonstrated to have increased tumourigenicity in mice animal models.

Increasing experimental evidence suggests that hypoxia and the HIFs regulate the sub-population of cancer stem cells. A recent study demonstrated that culture in hypoxia and activation of HIF1*α* expands the sub-population of cells positive for cancer stem cell marker, CD133 (Soeda *et al*, 2009). Cells under hypoxia also increased other markers associated with a more stem-like phenotype, such as CD44. At the same time, markers of neuron lineages (*β*3 Tubulin and GFAP) decreased in these cells. A study from Pahlman and colleagues (Jogi *et al*, 2002) has also shown that hypoxia in neuroblastoma cells can alter gene expression to that of a more immature phenotype. These data suggest that the fraction of a tumour considered to be cancer stem cells is plastic depending upon microenvironmental signals such as hypoxia. It is further hypothesised that the cancer stem cell phenotype may be a cell state induced in cancer cells depending on external and internal signals (Figure 2). The plasticity of the cancer stem cell state could have far reaching implications in how treatment of solid tumours is approached. Several groups have recently published data demonstrating this critical, and previously unknown, role of hypoxia in maintaining the stem-like fraction of cancer cells (Jogi *et al*, 2002; Heddleston *et al*, 2009; McCord *et al*, 2009).

McCord *et al* (2009) showed that hypoxia not only increased the fraction of CD133-positive cells, but also enhanced the stem-like phenotype of cell lines. By evaluating *in vitro* measures of the cancer stem cell phenotype, such as tumoursphere formation and stem cell marker expression, they showed that the stem-like population was enhanced. Using microarray techniques, they also determined that other genes related to stem cell functions, Sox2 and Oct4, were also increased in cells under hypoxia. It is interesting to note that these data showed genetic changes occurring at 7% oxygen where only HIF2*α* is known to be stabilized. This suggests a specific role of HIF2*α* in the plasticity of phenotype in cancer cells. Pahlman and colleagues (Pietras *et al*, 2008) demonstrated that expression of HIF2*α*, specifically, denotes neuroblastoma and breast cancer cells with a more immature phenotype and the presence of HIF2*α*-positive cells correlates to poor patient outcome. Data from the National Cancer Institute's REMBRANDT database supports the findings of McCord and Pahlman, in which patients with higher expression of HIF2*α* mRNA had lower survival probability (REMBRANDT Database of the National Cancer Institute, accessed 2 September 2009). Recently, it has been demonstrated that hypoxia is responsible for altering cellular phenotype by increasing proliferation, self-renewal, and the upregulation of stem genes in both stem and non-stem cancer cell populations (Figure 2A). Specifically, the HIF2*α* component of the hypoxic response can increase the expression of stem cell-associated genes and confer tumourigenic potential to non-stem cells (Heddleston *et al*, 2009). HIF2*α* transcriptionally regulates Oct4 (Covello *et al*, 2006). In addition, Oct4 and Nanog are part of the unique genetic signature of cancer cells (Ben-Porath *et al*, 2008; Ji *et al*, 2009). Together these studies outline a more sophisticated function of HIF2*α* outside of the canonical hypoxic responses. By regulating pathways associated with normal stem cell pluripotency, HIF2*α* may alter basic genetic activity of cancer cells to a more stem-like phenotype depending on the constantly fluctuating state of oxygen in the tumour. These findings are significant in showing that not only is HIF2*α* a critical part of the hypoxic response in cancer stem cells, but that it can promote a more stem-like phenotype in stem and non-stem cancer cells.

## Discussion

Tumour cell heterogeneity is currently modelled in two paradigms: stochastic and hierarchical models. The stochastic model posits that most cells within a tumour possess the ability to self-renew to maintain phenotypic copies of themselves. In addition, many phenotypically distinct cell populations in the tumour are able to recapitulate the primary tumour in a secondary location. The hierarchical model suggests that cells within a tumour are organised in a hierarchy, similar to normal stem cells. At the top of the hierarchy are cancer stem cells, which are the only cells that are able to self-renew and propagate the tumour. The other cells in the hierarchy are phenotypically distinct from the cancer stem cell and do not possess stem-like characteristics. However, these present models do not completely fit what is known about cancer stem cell biology, especially with the recent demonstration of HIF2*α* promoting a more stem-like phenotype in cancer cells. In order to better understand tumour biology, new approaches to how cancer cells form the tumour are needed.

**Figure 3:**
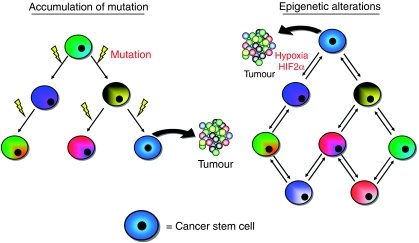
A new paradigm in modelling tumour biology. Classically cancer cell biology has been modelled in a stochastic or hierarchical sense. These models focus on which cells within a tumour population are responsible for tumourigenicity, but treat cellular progression an irreversible process: either all (or a large majority) can form tumours even as the cell phenotype may change, or that only the parental cells of the hierarchy (cancer stem cells) can form tumours, whereas their differentiated progeny act as a support structure. A new way to view the cellular organisation of tumour is to focus on how a cell may acquire the ability to be tumourigenic, rather than what definitive cell population forms the tumour. The mutation model posits that the ability to form tumours is because of an accumulation of specific mutations that allow the cells to evade therapy and growth more rapidly. In this model, each mutation is irreversible and the accumulation of important mutations (e.g. p53, EGFR) allows for a sustained sub-population of cancer stem cells. The second model describes the cellular structure of the tumour to be more plastic and allow for cellular adaptation to the microenvironment. The cancer stem cell sub-population drives tumour growth, but this population arises from adaptation of the cells to their microenvironment. External influences, such as hypoxia, can drive a reversible phenotype that can enhance stem-like properties of cells to ensure survival of the tumour.

The cells that comprise a tumour can instead be modelled as accumulation of mutations or alterations in epigenetic regulation (Figure 3). In the first proposed model, as cancer cells grow and divide they acquire new mutations, which give them distinct growth and survival advantages over previous generations. This can be observed in secondary tumours. Tumours that recur following treatment and resection (secondary tumousrs) are typically more resistant to further treatment and have a more aggressive phenotype. The increase in aggressive phenotype could be because of the cancer cells that acquired enough genetic mutation to survive treatments. However, the other aspect to this model is the genetic changes that occur are irreversible and do not allow for phenotype plasticity in the cancer cell population. A different way tumour biology could be modelled is by alterations in epigenetic regulation. Cells considered to be ‘cancer stem cells’ may have a less restricted epigenetic structure, such as loss of Histone 3 methylation, and not contain any new genetic mutations compared with the rest of the tumour. The lower genetic restriction allows the cell access to a wider variety of genetic transcripts and survive injury that may be sustained following patient treatment. As cells become more repressed by epigenetic silencing (DNA and histone methylation), they can become more differentiated and exhibit less stem-like behaviours. It is important to note that this model of tumour biology does not exclude the possibility of plasticity in the cellular phenotype. Recent experimental evidence supports the theory that ‘stemness’ may be regulated by the microenvironment. Several groups have shown that hypoxia can regulate the activity of histone demethylases (Xia *et al*, 2009). Differential histone methylation is known to be characteristic of embryonic stem cells and this suggests that hypoxia may effect the cancer cell phenotype by altering the epigenetic structure (Pan *et al*, 2007). The implications of this unique role of hypoxia in cancer emphasise the gathering evidence that hypoxia is an under-studied and under-appreciated factor in regulating cell fate.
